# Experiencing affective music in eyes-closed and eyes-open states: an electroencephalography study

**DOI:** 10.3389/fpsyg.2015.01160

**Published:** 2015-08-07

**Authors:** Yun-Hsuan Chang, You-Yun Lee, Keng-Chen Liang, I-Ping Chen, Chen-Gia Tsai, Shulan Hsieh

**Affiliations:** ^1^Department of Psychology, College of Medical and Health Science, Asia University, Taichung, Taiwan; ^2^Department of Psychiatry, College of Medicine, National Cheng Kung University, Tainan, Taiwan; ^3^Institute of Allied Health Sciences, College of Medicine, National Cheng Kung University, Tainan, Taiwan; ^4^Cognitive Electrophysiology Laboratory, Department of Psychology, National Cheng Kung University, Tainan, Taiwan; ^5^Department of Psychology, National Taiwan University, Taipei, Taiwan; ^6^Institute of Applied Arts, National Chiao Tung University, Hsinchu, Taiwan; ^7^Graduate Institute of Musicology, National Taiwan University, Taipei, Taiwan; ^8^Neurobiology and Cognitive Science Center, National Taiwan University, Taipei, Taiwan

**Keywords:** alpha asymmetry, electroencephalography, emotional valence, eye state, music

## Abstract

In real life, listening to music may be associated with an eyes-closed or eyes-open state. The effect of eye state on listeners’ reaction to music has attracted some attention, but its influence on brain activity has not been fully investigated. The present study aimed to evaluate the electroencephalographic (EEG) markers for the emotional valence of music in different eye states. Thirty participants listened to musical excerpts with different emotional content in the eyes-closed and eyes-open states. The results showed that participants rated the music as more pleasant or with more positive valence under an eyes-open state. In addition, we found that the alpha asymmetry indices calculated on the parietal and temporal sites reflected emotion valence in the eyes-closed and eyes-open states, respectively. The theta power in the frontal area significantly increased while listening to emotional-positive music compared to emotional-negative music under the eyes-closed condition. These effects of eye states on EEG markers are discussed in terms of brain mechanisms underlying attention and emotion.

## Introduction

In most human cultures, the use and function of music are closely related to its emotional impact on listeners. Recently, functional magnetic resonance imaging (fMRI) has provided the opportunity to observe neural correlates of different emotions evoked by music. Accumulating evidence indicates that joyful music activates the mesolimbic and mesocortical pathways, which plays a key role in motivation and reward-related behavior (Blood and Zatorre, 2001; Sloboda, 2001; Menon and Levitin, 2005; Salimpoor et al., 2011, 2013). On the other hand, fear-inducing music has been found to increase activity in the amygdala (Lerner et al., 2009) and its functional connectivity with the visual cortex and the superior parietal lobule suggests that it may heighten visual alertness (Koelsch et al., 2013). These studies showed that different neural and cognitive mechanisms underlie the processing of positively-valenced music and negatively-valenced music.

In addition to fMRI, electroencephalographic (EEG) measures have also been used to study the emotional processing of music. Earlier studies have reported that an EEG index, frontal alpha asymmetry, is related to emotions and approach/withdrawal motivational processes (Tomarken et al., 1992; Sutton and Davidson, 2000; Allen et al., 2001; Schmidt and Trainor, 2001; Pizzagalli et al., 2005). This index was found to reflect both emotional valence of the stimuli and affective style of an individual (Davidson, 2000; Perez, 2001; Glascher and Adolphs, 2003). The frontal alpha activity was more right-biased in response to positive emotions than to negative ones (Davidson, 1995; Oakes et al., 2004).

To examine the relationship between resting frontal alpha asymmetry and emotional reactions to musical stimuli (Schmidt and Hanslmayr, 2009), measured participants’ resting frontal alpha asymmetry and then presented emotional music excerpts. Their results showed that left-active individuals with greater alpha power over the right frontal electrode during the resting state tended to rate all stimuli as more positive compared to those with greater alpha power over the left frontal electrode during the resting state. These left-active individuals enjoyed more even when they listened to negative music.

The frontal activity associated with emotional processing may affect neural activity in the temporal and parietal lobes. The neural activity induced by music excerpts was examined by comparing pleasant and unpleasant states (Flores-Gutierrez et al., 2007), the results suggest that processing of emotions evoked by music involved sensory and cognitive integration. In listening to pleasant music, coherent activity in the upper alpha bands took place in most left hemisphere electrodes, indicating a functional coupling among some of the activated regions identified by fMRI, including the left primary auditory area, posterior temporal, inferior parietal and prefrontal regions. Moreover, in comparing the EEG activity of emotional processing of complex auditory stimuli using emotional excerpts and environmental sounds, Altenmuller et al. (2002) found a widespread bilateral fronto-temporal activation, left temporal activation was increased by positive emotional music and right fronto-temporal activation by negative emotional ones. Given that the left cortical network is involved in pleasant feelings, the modulatory effect of emotion valence on alpha asymmetry may be exhibited in both the frontal and posterior regions. Although alpha asymmetry in temporal (Davidson et al., 1990; Jones and Fox, 1992; Park et al., 2011; Lindquist et al., 2012) and parietal (Bruder et al., 2007; Stewart et al., 2011b) was reported to reflect emotional valence, no attempts have been made to examine the alpha asymmetry using musical stimuli. Different modes and tempi of music were used to investigate the EEG power asymmetry and emotional valence was found to be modulated by the mode (Trochidis and Bigand, 2013). In addition, emotional arousal was associated with tempo, in which an increased frontal activation was induced by faster tempo, especially in the left hemisphere.

Previous studies reported that the frontal, parietal, and temporal alpha asymmetries could reflect the emotional valence of stimuli; we therefore examined the alpha asymmetries at the electrode pairs of F3/F4, F5/F6, C3/C4, C5/C6, and T8/T7 (Figure 5). We calculate the alpha asymmetry index (AI) with the formula of (Schmidt and Hanslmayr, 2009):

(1) $$AI=\frac{P_{right}-P_{left}}{P_{baseline}}$$

*P_baseline_* is the average of alpha waves from all electrode sites of both the left and right hemispheres. According to Equation (1), a positive AI is associated with a right-biased alpha rhythm distribution. Given that neural activity is negatively correlated with alpha power, we expected that the larger *AI* would be found in the positive emotional condition relative to the negative emotional condition.

Theta wave power at the frontal midline (F area) is another EEG parameter related to music-induced emotion. Increased frontal midline theta power was found during exposure to positively valenced music relative to neutral or negative-valence music (Aftanas and Golocheikine, 2001; Lin et al., 2010; Salimpoor et al., 2011). Modulations of the frontal midline theta power have been linked to the cerebral metabolism in the anterior cingulate cortex (ACC; Pizzagalli et al., 2003), which is part of the limbic system engaged in emotional and attentional mechanisms (Davis et al., 1997; Asada et al., 1999; Khalfa et al., 2005; Mitterschiffthaler et al., 2007).

Although EEG indices such as alpha asymmetry and theta power have been employed in studying music-induced emotions, previous studies have not taken into account the possible effects of eye states. In some prior studies, participants were instructed to keep their eyes closed (Flores-Gutierrez et al., 2007; Sammler et al., 2007; Lin et al., 2010), but in others listeners were asked to open their eyes (Baumgartner et al., 2006; Schmidt and Hanslmayr, 2009) while listening to affective music stimuli. Of note, a global reduction in the resting alpha power has been found from the eyes-closed to eyes-open condition (Barry et al., 2007, 2009; Fonseca et al., 2013). Eyelids, among other muscles in the orofacial area, regulate our social engagement and modulate intake of sensory features in the social environment. Eye states may alter listeners’ emotional experiences. In the eyes-closed state, listeners’ negative emotion and arousal level evoked by fear-inducing music may be enhanced, as increased activation in the amygdala, anterior hippocampus, temporal pole and ventromedial prefrontal foci has been found in response to music that evokes fear (Lerner et al., 2009).

The purpose of this study was to examine how eye states modulate the association between musical emotions and EEG markers. In real life, listeners may have different emotions evoked by music in relation to different eye states. Different levels of arousal and attention associated with eyes-open and eyes-closed conditions may affect EEG indices. For example, the resting parietal alpha asymmetry under the eyes-closed state was reported as a risk factor for depressive disorder (Bruder et al., 2007), and comorbidity with anxiety disorder was characterized by left-biased brain activity (Stewart et al., 2011a). In addition (Boytsova and Danko, 2009), demonstrated an association between theta power and switching involuntary attention from internally-directed attention specific to the eyes-closed state to externally-directed attention specific to the eyes-open state. The present study examined whether frontal, temporal, and parietal alpha asymmetries, as well as the frontal midline theta power, could reflect music-induced emotion valence under different eye states. We hypothesized that the alpha AI calculated on the parietal sites may reflect emotional valence in the eyes-closed state because participant’s negative emotion induced by music may be exaggerated with their eyes closed. We also hypothesized that the alpha AI calculated on the temporal sites may reflect emotional valence in the eyes-open state but not in the eyes-closed state, because the increased baseline of alpha power under the eyes-closed state could largely reduce the AI values for the temporal regions. We also hypothesized to find an association between the frontal midline theta and positively valenced music.

## Materials and Methods

### Ethic Statement

This study has been conducted according to the principles expressed in the Declaration of Helsinki and the protocol was approved by the National Chung Cheng University Institutional Ethical Review Committee (IRB). All participants signed an informed consent form before participating in the experiments.

### Participants

Participants were 30 right-handed undergraduate students (mean age 21.6 ± 2.06 years: 20 males, average age 21.9 ± 1.92 years; 10 females, average age 21.2 ± 2.34 years) from National Cheng Kung University. No history of neurological or psychiatric disorders or cardiovascular diseases was detected in their self reports. Each participant was required to abstain from caffeine and tobacco use for 24 h before testing, and was paid NT $500 (US $15) for completing this experiment.

### Experimental Stimuli

Our study formed part of the project “Taiwan Corpora of Chinese Emotions and Biobehavioral Data,” and the present study used stimuli from the corpora of music-related emotions. Here we briefly mention the establishment of these corpora. Readers interested in details are referred to the original paper (Chen et al., 2013). Three musicians (two majored in composition and one in the French horn) selected 240 music excerpts from approximately 3000 commercial CDs. These excerpts are 20–30 s in duration and tend to convey one of the 20 emotion categories in the Geneva Emotion Wheel (GEW; see Scherer, 2005), including involvement/interest, enjoyment/pleasure, pride/elation, happiness/joy, enjoyment/pleasure, tenderness/feeling love, wonderment/feeling awe, feeling disburdened/relief, astonishment/surprise, longing/nostalgia, pity/compassion, sadness/despair, worry/fear, embarrassment/shame, guilt/remorse, disappointment/regret, envy/jealousy, disgust/repulsion, contempt/scorn, and irritation/anger. None of these 240 excerpts contain lyrics, and each excerpt has been rated by more than 1000 participants (aged 12–22 years) for its emotion category and emotional intensity. For this study, we selected ten 30-s music excerpts from the aforementioned corpora of music emotions. Four excerpts conveyed strong positive emotions, including two for happiness/joy and two for enjoyment/pleasure. Four excerpts conveyed strong negative emotions, including one for sadness/despair, one for worry/fear, and two for irritation/anger. These eight excerpts have been rated as conveying the highest or the second highest emotion intensity in these emotion categories. In addition, we selected two excerpts conveying weak sadness/despair and weak enjoyment/pleasure. As they were rated as conveying the lowest emotion intensity in these emotion categories, these two excerpts were used to prevent individuals from continuing to feel intense (positive or negative) emotional states in the present study. In addition, Davidson et al. (1990) emphasized that at least two emotions and a baseline condition must be compared, the two neutral musical excerpts in this study were used as the catch trials to help correct possibile guesswork of perceiving music excerpts conveying extreme emotions. Thus, the EEG measurements for these two excerpts were not analyzed in this study.

The positive and negative pieces differed in musical mode. All the positive pieces were in major modes. Three of the four negative pieces were in minor modes, and the remaining piece had no clear tonality. The positive and negative pieces did not significantly differ in tempo. The presentations of 10 music excerpts were counterbalanced using a Latin square design to avoid order effects.

### Experimental Apparatus

Electroencephalographic was recorded with 64-channel Neuroscan equipment (NeuroScan 4.3.1, USA), according to the international 10–20 system. A ground electrode was attached to the center of the forehead. Electrooculography (EOG) was measured to control for ocular artifacts. Vertical eye movement was measured using electrodes placed above and below the left eye, and horizontal eye movement was measured with electrodes placed lateral to the left and right external canthi. EEG and EOG signals were amplified by a multichannel biosignal amplifier (band pass 0.1–100 Hz) and A/D converted at 500 Hz per channel with 12-bit resolution, referenced to both mastoid electrodes. The recordings were re-referenced to the averaged mastoids in the final data averaging. The impedance of each electrode had to be less than 5 kΩ.

### Experimental Design and Procedure

Prior to the music listening session, each participant was given an emotional state questionnaire using Self-Assessment Manikin (SAM) developed by Lang (Lang et al., 2008) with 9-point scales to assess emotional valence, arousal and dominance at the beginning of the procedure. Next, participants did a simple cognitive task (60-s go/no go task) to maintain a neutral emotional state before listening to a music excerpt. The participants were asked to press the spacebar when they saw a target stimulus “S” on the screen and press no button when they saw a non-target “H” on the screen. Then 90-s baseline resting EEGs were recorded, with the participants’ eyes open or closed as assigned. These baselines were followed by the music listening session.

**FIGURE 1:**
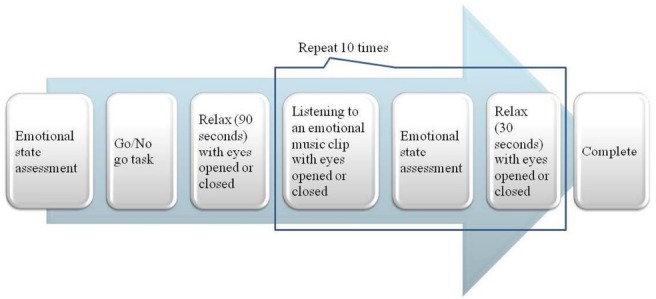
**Schematic description of experimental procedure**.

The music listening session consisted of two runs. Each run, consisting of 10 excerpts with different orders, was repeated twice. The participants were assigned to one of 10 counterbalanced orders with eyes open in one run and with eyes closed in the other run. The condition of eyes states was also counterbalanced. After listening to each music excerpt, the participants were asked to assess the emotion they experienced via SAM (Figure 1).

### Signal Preprocess and Data Analysis

The EEG signals between the 5 and 25th second intervals while listening to the music were analyzed. To remove the 60-Hz noise coming from the power line, the EEG signal was passed through a low-pass filter with a 50-Hz cutoff frequency. We used EEGLAB toolbox, an open-source toolbox for single-trial EEG analysis (Delorme and Makeig, 2004), to remove the eye movement component using an independent component analysis (ICA) routine. The EEG signal was transferred to the frequency domain via Fast Fourier Transform (FFT). Data were analyzed with a discrete Fourier Transform using a Hamming Window of the 5th to the 25th second interval and 75% overlap to lower the weighting and average the power. We focused on the theta band (4–7 Hz) and alpha band (8–12 Hz) for EEG indices calculation.

**FIGURE 2:**
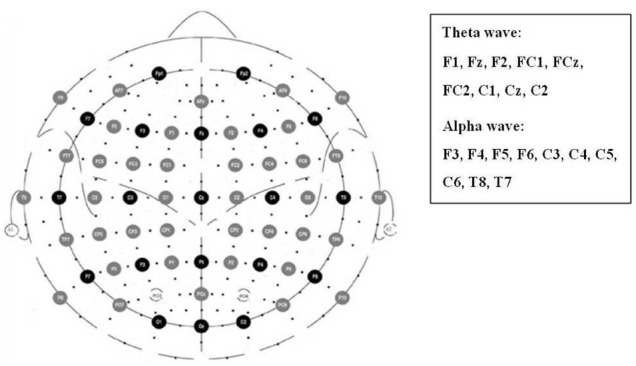
**Electrode sites for the analyses of frontal, parietal, and temporal alpha asymmetries as well as frontal midline theta power**.

Prior studies have demonstrated an association between frontal midline theta power and emotion valence (Sammler et al., 2007; Lin et al., 2010). The present study focused on the electrodes F1, Fz, F2, FC1, FCz, FC2, C1, Cz, and C2 for theta power calculation (Figure 2). The power change was calculated by natural logarithmic functions of baseline and emotional-eliciting power. Positive value indicated increased power. After estimating the theta wave of each electrode site, the nine electrodes were then averaged as the following three areas, F (F1/Fz/F2), FC (FC1/CF/FC2), and C (C1/Cz/C2).

### Statistical Analysis

To determine the different values of emotional valence between eyes-open and eyes-closed states, a two-factor analysis of variance with repeated measure design was conducted. In addition, several three-way ANOVAs with repeated measure design were used, with the emotional valence, eye conditions, and brain sites as independent variables, and the theta wave power of frontal midline (F/C) and frontal/temporal/parietal alpha asymmetry indices (AI) as dependent variables. *Post hoc* analyses were calculated using Tukey tests to compare functional connectivity patterns within the specific states. Moreover, association analysis was carried out to evaluate the relationship between participants’ rating of emotional valence and their resting *AI* (either at eyes-open or eyes-closed state) at frontal, temporal, and parietal areas.

## Results

### Emotional Valence Evaluation

**FIGURE 3:**
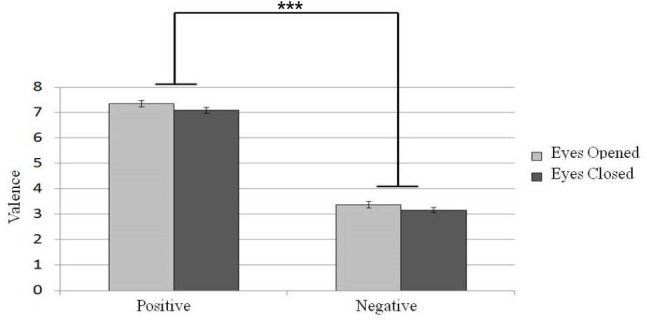
**Participants’ evaluation of emotion valence of different emotional music excerpts in different eye conditions.** Error bars represented as standard error of mean. ***p < 0.001.

The participants’ evaluation of different affective music excerpts under different eye states (eyes-open or eyes-closed) are shown in Figure 3. To determine whether the eye states would affect the evaluation of emotional valence under different emotional music conditions, a 2 × 2 repeated-measure designed two factor ANOVA (emotional valence with positive or negative and eyes states with eyes-open or eyes-closed) was conducted. We found a main effect of listeners’ report of emotional valence [*N* = 30, *F*(1,29) = 534.26, *p* < 0.001]. Taking the evaluation score as the comparison variable, we found that a higher score was given to positive emotion than to negative emotion. In addition, a main effect of eye states was found [*N* = 30, *F*(1,29) = 10.45, *p* < 0.01] such that listeners tended to rate the music stimuli more positively in the eyes-open state than in the eyes-closed condition. Therefore, listening to music under the eyes-closed state evoked more negative emotion.

### EEG-Based Functional Indices

**FIGURE 4:**
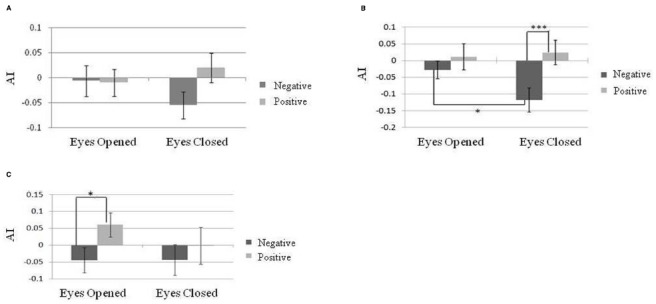
**Alpha asymmetry index (AI) during exposure to positively and negatively valenced music in different eye states on different brain areas. (A)** Frontal lobe. **(B)** Parietal lobe. **(C)** Temporal lobe. Error bars represented as standard error of mean. *p < 0.05; **p < 0.01; ***p < 0.001.

The baseline of temporal alpha power was significantly greater in the eyes-closed state than in the eyes-open state [*t*(29) = 9.25, *p* < 0.01]. A 3 × 2 × 2 repeated-measure designed three-way ANOVA, *AI* indices at different brain areas (frontal, temporal, and parietal electrodes), emotional valence (positive or negative), and eye states (eyes-open or eyes-closed) was carried out to determine whether *AI* indices could reflect emotional valence under different eye states. There was a significant main effect of *AI* indices on emotional valence [*N* = 30, *F*(1,29) = 6.52, *p* = 0.02] as well as an interaction among brain sites, emotional valence and eye states [*N* = 30, *F*(2,58) = 4.56, *p* = 0.02]. *Post hoc* comparisons showed that eye states had no effects on frontal *AI* either in response to positive or negative emotion (Figure 4A). However, we found a larger *AI* index on the parietal area in response to positive emotion than to negative emotion in the eyes-closed state [*N* = 30, *F*(1,174) = 11.31, *p* < 0.001; Figure 4B]. A similar result was found for the temporal *AI* index in the eyes-open state [*N* = 30, *F*(1,174) = 6.23, *p* = 0.01; Figure 4C]. Moreover, in the negative emotion condition, a larger *AI* index on the parietal area was found under the eyes-closed than under the eyes-open state [*N* = 30, *F*(1,174) = 4.03, *p* = 0.046]. Further analyses of the association between resting alpha asymmetry and emotional valence in either the eyes-open or eyes-closed state were not significant.

**FIGURE 5:**
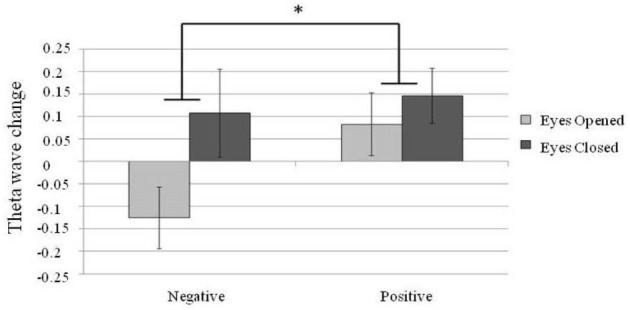
**Changes in the frontal midline (F area) theta power.** Error bars represented as standard error of mean. *p < 0.05.

For the theta power analysis, a 2 × 2 repeated measure design two factor ANOVA, theta power at different brain areas (frontal, temporal and parietal electrodes; emotional valence with positive or negative and eyes states with eyes-open or eyes-closed) was conducted. A main effect was found for brain area [*N* = 30, *F*(2,58) = 3.82, *p* = 0.03] and eye states [*N* = 30, *F*(1,29) = 7.59, *p* = 0.01]. A larger theta power was found in the eyes-closed state than in the eyes-open state. In addition, an interaction between brain areas and emotional valence [*N* = 30, *F*(2,58) = 4.31, *p* = 0.02] was found. *Post hoc* comparisons showed a significantly larger theta power in the F area when listening to positive emotion than to negative emotion [*N* = 30, *F*(1,87) = 4.66, *p* = 0.03; Figure 5].

## Discussion

Previous EEG studies on music perception have demonstrated an association between brain activation patterns and music-induced emotions. However, the possible impacts of eye states have been not systematically investigated. The frontal, temporal, and parietal alpha asymmetries as well as the frontal midline theta power under different eye states were examined in the present study. We found that the parietal and temporal alpha asymmetries correlated with emotional valence under the eyes-closed and eyes-open states, respectively. The results support Kallinen’s (2003) findings showing that an eyes-closed state would improve the concentration on music listening and modulate electrocortical activity in all cortical regions. Compared to negative = valence music, positive-valence music elicited more right-biased alpha power and greater frontal midline theta power in the listeners.

The right-biased alpha rhythm distribution elicited by positive-valence music excerpts was found in the frontal, temporal, and parietal lobes. This finding suggests some coherence between regions in the same hemisphere. In a combined EEG–fMRI experiment, a left cortical network including the posterior temporal-parietal and middle prefrontal regions were activated in perceiving positive-valence music but not in perceiving of negative-valence music. In addition, our findings are consistent with Altenmuller et al.’s (2002) finding of a widespread bilateral fronto-temporal activation accompanied by emotional attributions: an increased left temporal activation was accompanied by positive emotional music and right fronto-temporal activity was accompanied by negative emotional attributions. Coherent activity in the upper alpha bands occurred between most left hemisphere electrodes with a nodal focus in the temporo-parietal region, indicating a functional coupling among some of the activated regions identified by fMRI (Flores-Gutierrez et al., 2007). In our opinion, the co-activation of the left hemispheric regions during perceiving of positive-valence music may cause the right-biased alpha power in the frontal, temporal, and parietal lobes.

In the present study, the association between frontal alpha asymmetry and emotional valence was not significant, while that between the temporal/parietal alpha asymmetry and emotional valence was. The lack of association between frontal alpha asymmetry and emotional valence was consistent with some prior music-listening studies (Baumgartner et al., 2006; Sammler et al., 2007). While our findings appear to be inconsistent with the notion of frontal alpha asymmetry for emotional activation, it should be noted that a majority of studies reporting significant association between frontal alpha asymmetry and emotion valence used static affective stimuli such as images instead of dynamic ones such as music. Anterior temporal regions (amygdala, hippocampus, parahippocampus, and temporal pole), the temporal lobe ROI in our study, were found to respond to affective music (Blood et al., 1999; Baumgartner et al., 2006; Lerner et al., 2009). It has been suggested that the right amygdala is sensitive to dynamic emotional stimuli, while the left amygdala may decode the emotion in specific, sustained stimuli (Wright et al., 2001; Glascher and Adolphs, 2003). The dynamic emotional stimuli used in our study may have a different impact on the right and left amygdala, thereby modulating the temporal alpha asymmetry. This view is in line with the finding that the emotion valence of dynamic stimuli such as movie clips was associated with the temporal alpha asymmetry (Davidson et al., 1990; Jones and Fox, 1992; Park et al., 2011; Lindquist et al., 2012).

The association between the temporal alpha asymmetry and emotional valence was significant in the eyes-opened state but not significant in the eyes-closed state. The baseline of alpha power under different eye states was likely responsible for this result. Previous studies have demonstrated significantly increased alpha power in the eyes-closed state (Barry et al., 2009). We did replicate previous findings showing greater temporal alpha power in the eyes-closed state than in the eyes-open state. We concluded that the AI values for the temporal regions became too small and cannot reflect emotion valence under the eyes-closed state because of a higher baseline of alpha power.

The parietal alpha asymmetry was found to reflect emotional valence in the eyes-closed state. Figure 4B shows the smaller alpha asymmetry in the eyes-closed compared to the eyes-open state while listening to negative-valence music. This result may stem from participants’ experiencing stronger feelings of negative emotion with their eyes closed. This view was supported by participants’ evaluation of the emotional stimuli; their experienced emotion valence was more negative when they listened to music with their eyes-closed. Lerner et al. (2009) demonstrated that the eyes-closed state enhanced the negative emotion and arousal level during exposure to affective music, and a greater amygdala response to negative-valence music was found with closed eyes compared to open eyes. They therefore concluded that specific styles of attending can modify the activation of the amygdala in response to music. In our study, the left-biased parietal alpha power associated with negative-valence music may be related to the altered levels of arousal and attention as a result of increased amygdala activity. A previous study (Koelsch et al., 2013) found increased functional connectivity between the amygdala and the visual cortex along with superior parietal lobule in listening to fear-evoking music with eyes closed. The association between the parietal alpha asymmetry and emotion valence found in the present study may be attributed to the elevated arousal/attention level when listening to negative-valence music with eyes closed. This view is also supported by recent imaging studies on depressed patients, whose parietal alpha asymmetry was found to predict their mood state, and this association was modulated by caffeine through the influence of arousal (Stewart et al., 2011b).

Regarding the theta power changes, previous studies have demonstrated a greater frontal midline theta power when listening to positive-valence music compared to neutral or negative-valence music (Sammler et al., 2007; Li, 2010). The present study replicated this finding and further shows that modulation of frontal midline theta power by emotion valence is more prominent in the eyes-closed state (Figure 5). Modulation of the frontal midline theta power has been linked to the activity of the ACC (Pizzagalli et al., 2003), which is involved in emotional and attentional mechanisms (Davis et al., 1997; Asada et al., 1999; Khalfa et al., 2005; Mitterschiffthaler et al., 2007).

Although the present study suggests the temporal and parietal alpha asymmetries are useful indicators of emotion valence, it should be noted that our findings seem to contradict some prior studies. For example, Park et al. (2011) reported a significant decreased alpha power at the left temporal lobe during watching negative-valence videos; Bruder et al. (2007) found decreased alpha power on the left parietal site relative to the right parietal site in participants at risk for depression. In our opinion, our results disagree with previous findings for the following reasons. First, we used musical stimuli instead of videos, and the nature of the stimulus may affect the asymmetry of alpha power in the listeners/watchers. Second, the neural activity associated with negative emotions induced by music may differ from that associated with a depressed state. Further investigations are needed to shed light on these issues.

In summary, we found temporal/parietal alpha asymmetry and the frontal-central theta power can reflect listeners’ emotional state during music presentation. In addition, we demonstrated how the eye states modulate these EEG markers of emotion valence. The subjective emotional rating was affected by the eyes states; that higher valence was given to the music while the eyes are opened. In addition, under the eyes-closed state, parietal alpha asymmetry wave was larger to the positive music than to the negative music; but the temporal alpha asymmetry was more sensitive to the emotional music under eyes-open state. Moreover, the modulation of frontal midline theta power by emotion valence is more prominent under the eyes-closed state. Familiarity was suggested to enhance some level of experienced pleasure while prior exposure to music and increase electrodermal activity, but not significant (van den Bosch et al., 2013). The fact that familiarity was not measured in this study might be a limitation. Further investigation of the association between familiarity and eyes states while listening to music is needed. Although measures of emotional responses appear to be rated along with dimensions (e.g., valence and arousal) suggested by Mauss and Robinson (2009) rather than discrete emotional states (e.g., sadness, fear, anger), we believe that the current finding can be considered as a pioneering study that investigates the emotional valence and in patterns of EEG-based functional connectivity under different eyes states. Nevertheless, caution should be exercised about generalizing the results of this study because there were only four musical excerpts for each emotional category (positive or negative emotion). Future research using more excerpts for each emotional category may provide stronger evidence for the EEG markers for the emotional valence of music.

As music has been used as a tool of mood regulation in everyday life or clinical situations, the psychological effects of eye states may be taken into consideration. It was reported that using EEG alpha–theta brainwave training and relative relaxation therapies reduced alcoholics’ symptoms of depression and personality variable scores as well. In most clinical therapy protocols, patients/clients are required to close their eyes while receiving relaxation or biofeedback training (Saxby and Peniston, 1995). Our results further demonstrate that eye states influence the temporal and parietal alpha asymmetries through different mechanisms, and these results may have the potential for future real-life applications with dry and wireless EEG systems.

## Author Contributions

YC wrote the draft of this manuscript and conceptualized the data analyses with YL and SH. YL collected the data from participants and performed the statistical calculations. SH designed this study with CT as well as KL and IC. This study was supervised under SL and CT.

### Conflict of Interest Statement

The authors declare that the research was conducted in the absence of any commercial or financial relationships that could be construed as a potential conflict of interest.
